# Protective and Regenerative Roles of T Cells in Central Nervous System Disorders

**DOI:** 10.3389/fimmu.2019.02171

**Published:** 2019-09-12

**Authors:** Frances L. Evans, Marie Dittmer, Alerie G. de la Fuente, Denise C. Fitzgerald

**Affiliations:** The Wellcome-Wolfson Institute for Experimental Medicine, School of Medicine, Dentistry and Biomedical Science, Queen's University Belfast, Belfast, United Kingdom

**Keywords:** central nervous system, adaptive immune system, CD4^+^ T cells, neurological disorders, regeneration

## Abstract

Pathogenic mechanisms of T cells in several central nervous system (CNS) disorders are well-established. However, more recent studies have uncovered compelling beneficial roles of T cells in neurological diseases, ranging from tissue protection to regeneration. These divergent functions arise due to the diversity of T cell subsets, particularly CD4^+^ T cells. Here, we review the beneficial impact of T cell subsets in a range of neuroinflammatory and neurodegenerative diseases including multiple sclerosis, Alzheimer's disease, Parkinson's disease, amyotrophic lateral sclerosis, stroke, and CNS trauma. Both T cell-secreted mediators and direct cell contact-dependent mechanisms deliver neuroprotective, neuroregenerative and immunomodulatory signals in these settings. Understanding the molecular details of these beneficial T cell mechanisms will provide novel targets for therapeutic exploitation that can be applied to a range of neurological disorders.

## The Adaptive Immune System

**Figure 1:**
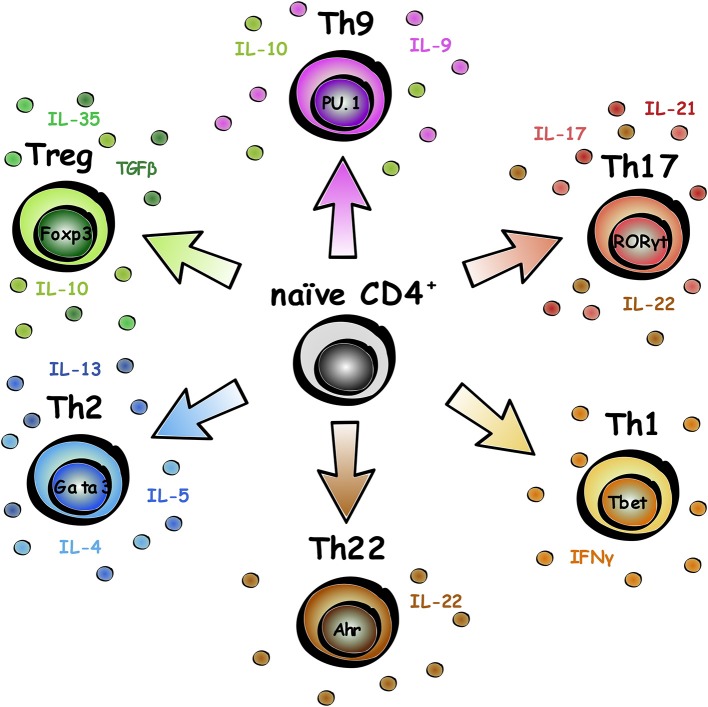
Subsets of CD4^+^ T lymphocytes. Naïve CD4^+^ T lymphocytes can differentiate into a range of subsets. The key transcription factor associated with the subset is labeled in the nucleus, and typical secreted factors listed around the cell.

The adaptive immune system is made up of B (for bone marrow-derived) and T (for thymus-derived) lymphocytes, that have evolved to protect us from pathogens and mount a faster immune response for repeat infections against the same pathogen (1). Naïve CD4^+^ T lymphocytes undergo differentiation into various subsets, specification of which can be influenced by expression of cytokines in the microenvironment as well as intracellular transcription factors (2). This review will focus on CD4^+^ T lymphocytes, and to a lesser degree, CD8^+^ T lymphocytes, and emerging regenerative roles of these cells in neurological disease. CD4^+^ T lymphocytes can be divided into different subsets based on biological roles, transcription factor expression, and cytokine release, as summarized in Figure 1. Subset functional roles and characterization are reviewed in more detail in Caza and Landas (3).

## CNS Homeostasis

Efficient CNS homeostasis is critical to overall health and anatomical barriers have evolved to ensure that the CNS is selectively protected from potentially harmful peripheral influences such as microorganisms, toxins, and even aberrant immune function. The choroid plexus acts as a blood-cerebrospinal fluid (CSF) barrier and is a selective gateway for leukocyte entry into the CNS (4). In the brain of healthy individuals, T cells are only present sporadically in the parenchyma and in the perivascular space (5). Approximately 150,000 T lymphocytes are present in the CSF of healthy individuals (6) to carry out immune surveillance of the CNS and deep cervical nodes (7). In collaboration with CNS-resident immune cells, this selectivity of peripheral immune cell trafficking ensures that the CNS is afforded sufficient immune protection without being subjected to potentially harmful inflammatory responses on a regular basis.

## T cells in Brain Development and BEHAVIOR

Hippocampal neurogenesis is dependent on the adaptive immune system, and is significantly impaired in severe combined immune deficiency (SCID) mice that lack T and B lymphocytes due to a *Rag1/2* gene deletion (8). Neurogenesis impairment was found to be dependent on CD4^+^ lymphocytes, as transplantation of CD8^+^ lymphocytes into *Rag2*^−/−^ mice (lacking T and B cells) did not rescue the effect in the same manner (9). Interestingly, mice with a T cell receptor against endogenous myelin basic protein (MBP) showed enhanced hippocampal neurogenesis and improved spatial learning in comparison to mice with a T cell receptor to non-self antigen (8).

*Rag*2^−/−^ mice showed impaired learning/memory performance in the Morris water maze (MWM) task, but this impairment was not attributable to B cell deficiency as B cell-deficient mouse (μMT mice) performance was comparable to controls. This impaired MWM performance was also evident in MHC II^−/−^ mice that are deficient in CD4^+^ T cells but have CD8^+^ T cells, suggesting that CD4 or MHC II functionality is required for normal learning and memory performance (10).

Further evidence for roles of T cells in neurodevelopment comes from studies of mice deficient in mature T cells, in both nude (lacking T lymphocytes due to a disruption in the *Foxn1* gene that causes deterioration of the thymus) and SCID mouse models. These mice had lower levels of brain-derived neurotrophic factor (BDNF), a higher number of cognitive deficits, and poor performance in the MWM. These deficits can be rescued in nude mice when T cells are repopulated by adoptive transfer from WT mice (11). *Rag*1^−/−^ immunocompromised mice show increased depressive and anxiety-like behavior that is rescued by transplanting CD4^+^ T cells, but not CD8^+^ T cells (12). Such findings are not restricted to murine models, as activated human T cells secrete bioactive BDNF that supports neuronal survival *in vitro* (13). Pharmacological loss-of-function studies have also provided evidence of a role for T cells in neurodevelopment. Removal of lymphocytes from the meningeal spaces in mice using fingolimod (sphingosine-1-phosphate receptor modulator) or anti-VLA4 [which attenuates the migration of T cells and monocytes across the blood brain barrier (BBB)] also resulted in impaired learning outcomes (14).

Taken together, these and a range of other studies have shown that the adaptive immune system plays important roles in CNS homeostasis and impacts behavior, but it is also very important in disease progression outcomes across neurological conditions. The regulatory T cell (Treg) subset of CD4^+^ T lymphocytes has been shown to play a regenerative role in several tissue types, such as the kidney, skin, retina, skeletal muscle, lung, myocardium, bone, and hair follicles [reviewed in (15) and (16)]. Given the described roles of T cells in the development of the CNS, and that many regenerative processes have similar biological mechanisms to development, it is not surprising that studies are emerging showing regenerative roles of T cells in the CNS in neurological disease.

## Amyotrophic Lateral Sclerosis

Amyotrophic Lateral Sclerosis (ALS) is an adult-onset neurodegenerative disease that is typically fatal within 3–5 years (17). Motor neurons in the motor cortex, spinal cord, and brainstem undergo cell death leading to loss of functions such as movement, coordination, and breathing. There are no disease-modifying treatments available that significantly alter or improve the course of the disease (17). ALS features neuroinflammation, but most emphasis in research has been on glial reactivity and the innate immune response (18). However, the influence of the adaptive immune system in ALS is gathering increasing attention; there are changes in the peripheral immune system and inflammatory markers that likely contribute to the pathology of the disease, but the relative importance of specific changes are yet to be fully determined (19).

A number of studies have reported increased numbers of T cells in the CNS of patients with ALS. T cell infiltrates were found in post-mortem CNS samples from ALS patients (20), and both CD4^+^ and CD8^+^ T cell subsets were observed in close proximity to degenerating neurons in the spinal cords of ALS patients (21). Interestingly, T cells isolated from the CSF of ALS patients appear to be clonally expanded, suggesting antigen-mediated activation in the CNS (22).

Reports of T cell populations in the peripheral blood of ALS patients remain controversial. Murdock et al. (19) found no significant difference in the number of CD4^+^ or CD8^+^ T cells compared to controls initially. However, disease progression correlated with decreased numbers of CD4^+^ T cells in the blood (19). In contrast, Mantovani et al. (23) reported elevated levels of CD4^+^ T cells in the peripheral blood of ALS patients in comparison to healthy controls. As such, the relative change in the peripheral T cell populations in ALS remains an open question.

In the SOD1 mutant mouse (SOD1mt), a model of familial ALS, lymphocyte infiltration into the CNS is observed, most prominently at later stages of the disease (24). SOD1mt mice crossed to *Rag2*^−/−^ mice (deficient of B and T lymphocytes) showed a similar timing of disease onset but accelerated disease progression. These findings were recapitulated in SOD1mt mice crossed with CD4^−/−^ mice (deficient of CD4^+^ T lymphocytes). In comparison to the SOD1mt mouse, both lymphocyte-deficient mouse models showed reduced microglial activation; reduced mRNA expression of the neurotrophic factors IGF-1, GDNF, BDNF, and of the glutamate transporters GLT-1 and GLAST; reduced IL-4, TGF-β, CX3CR1, and YM1 (M2-like marker); and elevated mRNA expression of TNF-α, IL-6, and NOX2. Following bone marrow transplantation, both CD4^+^ and CD8^+^ T cell populations infiltrated the spinal cord in SOD1mt mice. However, CD8^+^ T cells were only detected at the end stage of the disease, whereas CD4^+^ T cells were already detectable at P75. Many of the biological parameters described in the lymphocyte-deficient SOD1mt mice were reversed after bone marrow transplantation which reconstitutes lymphocyte populations (25, 26). A similar study reported comparable disease onset but accelerated disease progression in a SOD1mt mouse model lacking T cells (deficiency of functional T cell receptor (TCR) using TCRβ^−/−^ mice) (26). Together, these studies suggest that T cells may play a neuroprotective role in ALS through a variety of potential mechanisms.

Elevated numbers of Treg (CD4^+^CD25^+^Foxp3^+^) were detected in the blood of the SOD1mt mouse model from disease onset and were sustained throughout the disease course. Foxp3, IL-4, and IL-10 mRNA was elevated in spinal cords in early disease phases, while intensity of the Foxp3 signal decreased at later disease stages suggesting Foxp3 intracellular expression was reduced. mRNA expression of the Th1-associated transcription factor, T-bet and Th1-associated cytokine, IFN-γ, but not the Th2-associated transcription factor, GATA3, were also elevated in early disease stages, suggesting an increase of the infiltration of Th1 cells but not Th2 cells. When Treg were transferred to SOD1mt/*Rag2*^−/−^ mice, survival was significantly prolonged. This result correlates with disease progression data from human patients, as those ALS patients with lower numbers of peripheral Treg suffered a more rapidly progressing disease course in comparison to those with higher numbers of Treg (27).

The Treg subset of CD4^+^ T cells is frequently studied in inflammatory and degenerative disease, given the potency of these cells to inhibit the function of proinflammatory immune cells. Sheean et al. (28) reported an inverse correlation between levels of Treg and ALS disease progression and went on to show that Treg expansion in the SOD1G93A mouse slowed disease progression and augmented survival duration. A functional dysregulation of Treg was reported in ALS patients as demonstrated by reduced suppressive capacity when tested in responder T lymphocyte proliferation assays. ALS patients with rapid disease progression had an even more profound impairment in Treg suppressive function in comparison to ALS patients with slower disease progression, associated with reduced levels of Foxp3 mRNA expression, the canonical transcription factor of Treg. Lower Foxp3 mRNA correlated with greater disease burden and reduced survival. *In vitro* expansion of patient-derived Treg with IL-2 and rapamycin augmented the suppressive capacity of Treg (29), suggesting that Treg from ALS patients may be amenable to therapeutic modulation. A phase II trial of rapamycin is currently underway and the primary aim is to determine whether rapamycin treatment increases Treg numbers in treated patients compared with a placebo control group (30).

Another approach being taken is to administer Treg as cell therapy. In 2016, Alsuliman et al. (31) reported a method to isolate and expand good manufacturing practice-compliant Treg from ALS patients for clinical use. A Phase I clinical trial investigating treatment with infusions of autologous expanded Treg, alongside subcutaneous injection of IL-2, was carried out on three patients with ALS. The trial showed that this treatment was safe, well-tolerated and reported slowed progression of the disease, although as this was a Phase I trial, the low sample size must be noted. An increase of Treg numbers was observed after treatment and Treg suppressive function in peripheral blood was improved (32). The results from this trial provided the basis for a Phase II clinical trial with a randomized placebo-controlled group, as well as investigating treatment efficacy and safety over a longer time period, dose optimisation, and a larger study size. The Phase II trial is underway with the first patient recruited in June 2017 (MIROCALS: Modifying Immune Response and OutComes in ALS) (33).

**Figure 2:**
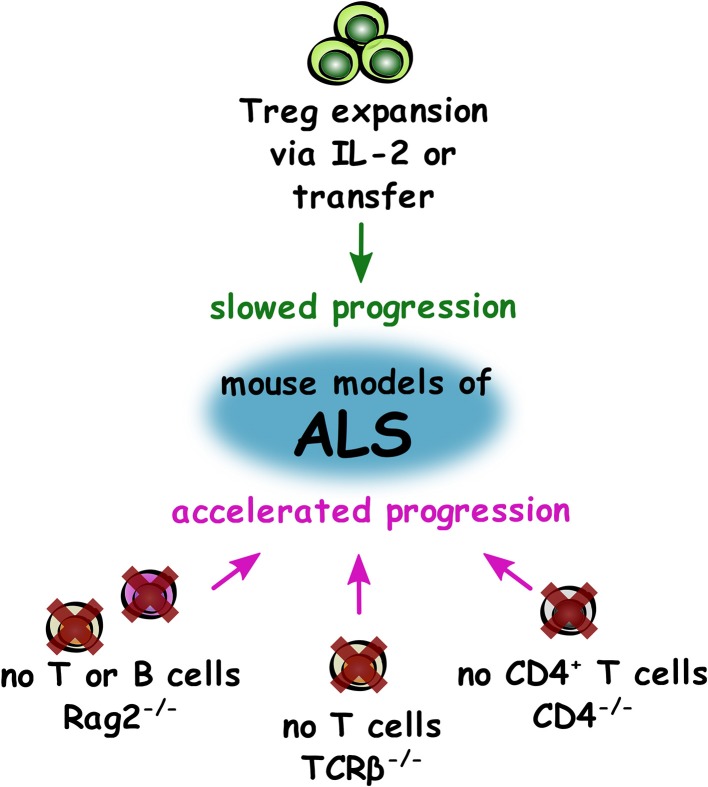
Impact of T cell supplementation and deficiency on disease progression in mouse models of ALS. Summary of changes to the adaptive immune system that either slow (green) or accelerate (magenta) progression in mouse models of ALS.

Overall in ALS studies (summarized in Figure 2), the most compelling evidence from both mouse models and in human disease is that Treg appear to provide a neuroprotective effect and slow disease progression. Therefore, autologous infusions of expanded Treg with subcutaneous injection of IL-2 may prove to be an effective disease-modifying treatment in the coming years if the current clinical trial shows positive results. The intriguing studies showing detrimental effects of total lymphocyte deficiency in models of ALS suggest that other T cells may also hold therapeutic potential and certainly warrant further investigation.

## Alzheimer's Disease

Alzheimer's disease (AD) is characterized by loss of neurons and synapses primarily in the cerebral cortex. This neurodegeneration causes memory loss, and in later stages can cause impairment in other capacities such as language, emotions, and behavior (34). Histopathology of AD brains shows the presence of protein aggregates in and around neurons: amyloid-beta (Aβ) plaques and neurofibrillary tangles that are aggregates of hyperphosphorylated tau (35). These features are thought to underlie neurodegeneration in AD.

Analysis of the peripheral immune system of patients with AD in comparison to healthy controls demonstrated a significant reduction of CD3^+^ T lymphocytes. There was no significant change in the CD4^+^/CD8^+^ ratio, however a slight increase of CD4^+^ T cells and slight decrease in CD8^+^ lymphocytes was observed (36). Research by Larbi et al. (37) also demonstrated differences in T lymphocyte numbers in AD patients compared to healthy controls. Significantly decreased proportions of naïve T cells were observed, with elevated numbers of memory cells and CD4^+^ T cells. CD4^+^CD25^+^ T lymphocytes, assumed potentially to be Treg, were also decreased in AD patients. A significantly increased Aβ-specific CD4^+^ T cell intrinsic reactivity was detected in blood samples from AD patients, however this was also observed in elderly subjects, suggesting that this T cell response was associated with the aging process in general rather than with AD specifically (38). Such findings highlight the challenges inherent in studying immune function in AD in the face of advancing age, which is already known to significantly impair the diversity and functionality of the immune system.

A number of post-mortem studies have confirmed the presence of T cells in the brains of AD patients (39, 40). Research by Merlini et al. (41) demonstrated an increased frequency of T cells in the brain in advanced stages of AD, and reported that all CD3^+^ cells were also CD8^+^ T cells (particularly in the hippocampus). The frequency of CD3^+^ extravascular T cells were found to correlate with tau pathology in the brains of AD patients, but not with the number of Aβ plaques. Smolders et al. (5) also observed white matter-associated T cells (primarily CD8^+^ T lymphocytes) in the brains of AD patients. Misfolded amyloid and tau can independently lead to T cell extravasation (42, 43), but what drives this T cell infiltration is unclear as T cells were not reported to be interacting with the plaques or tangles present in transgenic mouse models of AD. Two recent studies identified a proinflammatory T cell profile in the CSF of AD patients (44) and in amnestic mild clinical impairment, an early stage of AD (45). Interestingly, polymorphisms in genes associated with antigen presentation to T cells were identified as susceptibility loci for AD (46–48), lending further support to the potential relevance of T cells in AD.

Studies in murine models demonstrate that AD-susceptible mice, known as 5xFAD mice that express five human AD-linked transgenes in APP and PSEN1, have more rapid disease progression when their adaptive immune system is genetically ablated (*Rag2*^−/−^/*Il2r*γ^−/−^-5xFAD mice), suggesting a protective role for adaptive immunity in the diseased brain. Neuroinflammation is greatly increased in these mice, and the microglial phenotype is skewed, causing increased cytokine production and reduced phagocytic capacity. WT bone marrow transplantation into these immunodeficient mice resulted in a 47% reduction in plaque volume and significantly reduced this AD pathology (49), demonstrating a beneficial effect of lymphocyte reconstitution in this model.

On the contrary, APP/PS1 mice that express human transgenes for mutations in APP and PSEN1, crossed with lymphocyte-deficient *Rag2*^−/−^ mice showed less abundant fibrillar Aβ deposits and 25–30% less amyloid plaque pathology than the APP/PS1 mice at 8 months of age (50). This model also showed a decrease in GFAP immunoreactivity but similar Iba1 immunoreactivity suggesting reduced astrogliosis but no effect on microgliosis. Aged APP/PS1 mice (12 months) that were irradiated and then reconstituted with bone marrow from *Rag2*^−/−^ donors, showed a reduction in brain Aβ load but no changes in plaque number. Elevated plasma Aβ levels were reported together with increased microgliosis, and elevated numbers of plaque-associated Aβ phagocytosing microglia. These data suggest that the adaptive immune system modulates and dampens microglial responses to misfolded Aβ peptides (50).

The impact of T cell depletion has also been investigated in other mouse models of AD. In the Thy22-Tau mouse model of AD, which accumulate increased levels of human tau as they age and are used to study tau aggregation, T cells were depleted with a daily injection of anti-CD3 depleting antibody from 4 to 9 months of age and compared to WT controls. CD3^+^ depletion in WT controls caused no difference in their learning/memory behavior in the Y-maze task, whereas CD3^+^ depletion in Thy22-Tau mice rescued behavioral impairment to WT levels. T cell infiltration is not thought to be directly involved in tau proteinopathy (it does not change tau phosphorylation nor deposition), but suggests that T cell infiltration regulates inflammation through microglia and astrocytes as anti-CD3 treatment was linked to the downregulation of neuroinflammatory markers (43).

Proinflammatory T cells have a negative effect on AD pathology in mouse models of AD. APP/PS1 mice have significant infiltration of T cells in the brain, and a proportion of these cells secrete the proinflammatory cytokines IFN-γ or IL-17 (51). Adoptive transfer of Aβ-specific Th1 cells (polarized *in vitro*) into APP/PS1 mice increased microglial activation and Aβ deposition, and impaired cognitive function was reported, presumably as a result of these changes. These negative effects were attenuated by treatment with anti-IFN-γ antibody (51), suggesting that IFN-γ drove disease pathology in this model. Conversely, vaccine-induced Th1 cells with specificity for Aβ enhanced Aβ clearance, but caused encephalitis as a side effect (52), whereas Th2 cells specific for Aβ reversed cognitive decline and synaptic loss in AD-like pathology (53, 54). A clinical trial investigating Aβ vaccination (AN1792) was halted after the occurrence of meningoencephalitis in 6% of vaccinated patients. Of the 6% of patients that developed meningoencephalitis, 66% recovered in a number of weeks while the remainder had residual cognitive and neurologic impairment. The development of meningoencephalitis did not correlate with anti-Aβ antibody titration, suggesting that meningoencephalitis was not associated with a B cell response against Aβ, but rather a detrimental response of Aβ-specific T cells. Subsequent work in animal models of AD has shown similar side effects, with meningoencephalitis that was abrogated when animals were immunized with Aβ 1–42 in particular. The authors suggested that three conditions needed to occur simultaneously for the development of meningoencephalitis: presence of Aβ in the brain, a particular genetic background that will elicit a T cell response due to a high affinity for the Aβ T cell epitope and a pro-inflammatory signal (52). This may be enhanced by the release of antigenic peptides from Aβ processing that may trigger T cell inflammatory responses (55). These severe side effects were attributed to vaccine-induced T cell responses, suggesting a detrimental role of Aβ-specific T cells in the context of AD (55–57). However, taking into account the positive results of Aβ vaccines in mouse models of AD, further investigation of development of meningoencephalitis is required to determine the role of T effector cells in this severe adverse effect.

Research investigating whether T-cell associated cytokines could be of therapeutic value in AD has shown some controversial results. Administration of IL-4 and IL-10 improved AD symptoms in mice (58, 59) through enhanced neurogenesis, improved spatial learning and reduced Aβ deposition in APP/PS1 mice (60). However, two recent studies using TgCRND8, Tg2576, and APP/PS1 AD mouse models, show a detrimental role of IL-10 in AD pathology (61, 62). Peripheral IL-2 delivery was associated with elevated Treg in the brain of APP/PS1 mice, rescuing spatial memory impairment in the MWM and impaired synaptic plasticity, and restored spine density (63). Similarly, low dose IL-2 enhanced Treg frequency and restored cognitive functions, and enhanced the number of plaque-associated microglia (64). However, it is yet to be determined if this beneficial role is due to IL-2-mediated-Treg expansion, IL-2 acting directly on IL-2 receptors present on neurons, or both. The first possibility is supported by the fact that transient early Treg depletion using anti-CD25 antibody in an APP/PS1 AD mouse model accelerated the onset of cognitive deficits without influencing Aβ deposition (64). These results are supported by studies of Baek et al. (65), who adoptively transferred CD4^+^CD25^+^ Treg into 3xTg-AD mice and observed improved cognitive function, reduced deposition of Aβ plaques, decreased microglial activation, and decreased production of pro-inflammatory cytokines such as IL-6, IFN-γ, or IL-17. Similarly, matrine and bee venom phospholipase A2 have been shown to invert the Th17/Treg ratio and to increase Treg proportion in AD rat and mouse models respectively. This increase in Treg led to a decrease in Aβ plaques, a decrease in microglial and T cell infiltration in the brain, and led to cognitive improvements (66, 67). These studies support the view that there may be value in using T cell-associated cytokines or Treg adoptive transfer in the treatment of AD, but caution must be exercised, and the benefit of treatment may vary based on disease stage.

In contrast, other research has found Treg to have a negative effect on AD pathology. Depletion of Foxp3^+^ cells or pharmacological inhibition of Treg activity in 5xFAD mice induced clearance of Aβ plaques, mitigated the neuroinflammatory response, and reversed cognitive decline (68). In the 5xFAD mouse model, elevated levels of Foxp3^+^ cells are reported in the spleen which has also been described in AD patients (69, 70). Taking into account that Treg are one key source of IL-10 (Figure 1), these results are supported by the fact that IL-10 caused an increased accumulation of Aβ plaques and subsequently impaired memory function (61).

The previous Treg data were partially supported by a second study by the same group. Baruch et al. (71) treated 10-month-old 5xFAD mice with advanced diseased pathology with two intraperitoneal injections of an antibody against programmed cell death protein 1 (PD-1) (PD-1 is involved in suppressing T cell inflammatory activity) or an IgG control antibody. Mice treated with the PD-1 antibody had significantly increased IFN-γ producing CD4^+^ T cells and IFN-γ expression at the choroid plexus, as well as increased myeloid cells in the brain. PD-1 treatment reduced cognitive deficits and this reduction was maintained even 1 month post-treatment, together with reduced Aβ plaque load in the hippocampus and cortex and reduced astrogliosis. These effects were also replicated in APP/PS1 mice, supporting a neuroprotective role for CNS-specific cell mediated immunity. Even though these results are interesting and suggest potential novel treatments for AD, further validation by other groups is required.

Anti-inflammatory and immunosuppressive treatment strategies are proposed to be beneficial in AD as in other neurological conditions, however research to date has not yet produced consensus. Controversial and sometimes contradictory results following depletion of components of the adaptive immune system have emerged, which may correspond to differences in mouse models used and the timing of depletion in relation to disease onset. The differences regarding the role of Treg in AD may be due to differences in experimental design of the timing of Treg depletion: Dansokho et al. (64) depleted Treg earlier (5–6 weeks) at the start of amyloid deposition and gliosis, whereas Baruch et al. (68) depleted Treg at 4–5 months when neuropathology has developed significantly. Based on clinical trial data and inflammatory effects, modulating T cells for the treatment of AD may require improved specificity before it is safe to develop a therapeutic for humans, particularly if the divergent findings in mouse models are attributable to disease stage-specific pathogenic mechanisms.

## Parkinson's Disease

Parkinson's disease (PD) is a neurodegenerative disease characterized by the formation of abnormal protein aggregates inside neurons, termed Lewy bodies and Lewy neurites, and cell death of dopamine-secreting neurons in the substantia nigra in the brain. PD typically manifests with symptoms involving motor dysfunction, but neuropsychiatric symptoms can also occur (72). Immune alterations have been reported in PD and animal models of the disease, but the relative contributions of different immune system changes are not yet fully understood.

In PD, frequencies of lymphocyte subpopulations are altered in the periphery. Saunders et al. (73) reported decreased frequencies of CD4^+^ T cells in PD, and others have described a reduced CD4^+^:CD8^+^ ratio due to decreased proportions of T helper lymphocytes and increased proportions of cytotoxic T lymphocytes (74–76). PD patients exhibit a shift toward a Th1 immune response with increased IFN-γ, reduced numbers and suppressive capacity of Treg, reduced B lymphocytes, and an increase in NK cells (73–75, 77). CD8^+^ and CD4^+^ T cells, but not B cells, infiltrate the brain during Parkinson's disease (PD), based on evidence from post-mortem human tissue samples and the 1-methyl-4-phenyl-1,2,3,6-tetrahydropyridine (MPTP) mouse model of PD (78).

MPTP-induced dopaminergic cell death was markedly attenuated in the absence of mature T lymphocytes in both *Rag1*^−/−^ and *Tcrb*^−/−^ immunodeficient mouse strains (78). Similar attenuation of MPTP-induced dopaminergic cell death was observed in mice lacking CD4^+^ T cells as well as in *Rag1*^−/−^ mice reconstituted with FasL-deficient splenocytes (FasL was chosen as a target to investigate CD4^+^ T cell-mediated cytotoxicity). Mice lacking CD8^+^ lymphocytes and *Rag1*^−/−^ mice reconstituted with IFN-γ-deficient splenocytes were not protected, therefore supporting that T cell-mediated dopaminergic toxicity was almost exclusively arbitrated by CD4^+^ T cells and required the expression of FasL but not IFN-γ (78).

On the other hand, adoptive transfer of T cells reactive against Copolymer-1 [also known as glatiramer acetate, an immunomodulatory therapy used to reduce relapse rates in multiple sclerosis (MS)] into MPTP-intoxicated mice led to accumulation of T cells in the substantia nigra, suppression of microglial activation through secretion of IL-4 and IL-10 by T cells, and increased astrocyte-associated glial cell line-derived neurotrophic factor. This adoptive transfer of T cells resulted in increased survival of nigrostriatal neurons, and this effect was negated by depletion of the donor T cells (79). In particular, Treg are also able to protect nigrostriatal neurons via cell-to-cell contact between Treg and dopaminergic neurons by a CD47-SIRPα interaction which triggers Rac1/Akt signaling *in vitro* (80). Similarly, in the MPTP mouse model of PD, adoptive transfer of activated Treg resulted in an increased protection against neurotoxicity, and prevented microglial release of reactive oxygen species *in vitro*, leading to prevention of neuronal damage (81, 82). This result was also observed when the MPTP mouse model was treated with GM-CSF-differentiated bone marrow-derived dendritic cells (BMDCs). Adoptive transfer of BMDCs attenuated neuroinflammation and induced a significant increase in Treg numbers within the CD4^+^ T cell population leading to protection of dopaminergic neurons (83). A similar effect has also been observed in a small preliminary study performed to determine the effects of sargramostim (recombinant GM-CSF) in PD. Sargramostim was shown to protect against nigrostriatal neurodegeneration in rodent PD models (84, 85). In this preliminary study, Gendelman et al. (86) showed that sargramostatim increased the numbers of CD3^+^ and CD4^+^ T cells, increasing in particular the frequencies of CD4^+^CD127^lo^CD25^hi^ Treg as early as 2 weeks of treatment, which then remained elevated. Moreover, even though the study was not powered to evaluate the clinical outcome in motor activities, they observed an overall improvement of the treated patients when compared to the placebo control group, which appeared to be associated with alterations in the T cell profile and changes in the pro- and anti-inflammatory gene expression profile (86).

**Figure 3:**
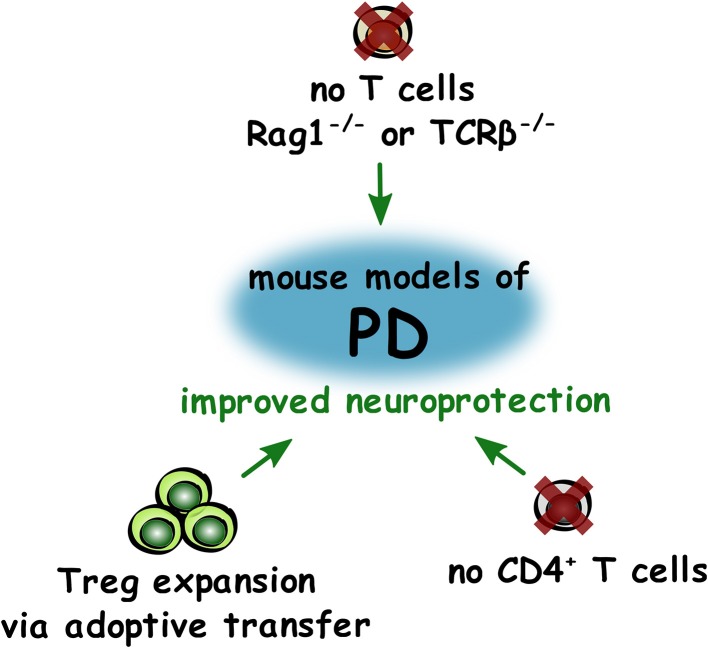
Roles of T lymphocytes in improving neuroprotection in PD. Summary of changes to T lymphocytes that led to improved (green) neuroprotection in mouse models of PD.

In conclusion, these studies appear to show that depletion of CD4^+^ T lymphocytes is beneficial in the MPTP model of PD, but this may be due to removal of the increased numbers of Th1 cells present. As adoptive transfer of Treg appeared to have neuroprotective effects, and a decrease in the numbers and suppressive capacity of Treg is impaired in PD, increasing the numbers of Treg may be beneficial for preserving dopaminergic neurons in PD (Figure 3).

## Multiple Sclerosis

Unlike previously discussed diseases in which the main driver of pathology is unexplained neurodegeneration, MS is an immune-mediated demyelinating disease of the CNS, causing symptoms such as visual deficits, fatigue, mobility issues, sensation issues, bladder and bowel problems, amongst others. New symptoms can be within isolated attacks known as relapses, or build up over time in progressive forms of MS. After a relapse, symptoms may resolve, but often neurological deficits remain typically in greater frequency as the disease advances (87). MS is the disease in which the greatest amount of T cell research in the CNS has been performed. Much of our knowledge of how T cells migrate into the CNS has been learned from studies of MS and through animal models of MS. The most commonly used animal model to study MS is experimental autoimmune encephalomyelitis (EAE) which is in fact, a range of models [reviewed in (88), (89)]. Although EAE studies have uncovered a number of pathogenic mechanisms of CNS tissue damage, these models have somewhat limited utility for studies of myelin regeneration as, in these models, T cells induce primary demyelination, axonal loss, and neurodegeneration. As such, distinguishing pathogenic and regenerative T cell roles is very challenging and there are reduced numbers of axons available to be remyelinated. Similarly, targeting of T cell migration and function has delivered therapeutic benefit to thousands of MS patients around the world and these patients, in turn, have helped to advance knowledge of neuroimmune functions of T cells.

A study comparing peripheral blood, CSF, and post-mortem brain samples (divided into normal appearing white matter (NAWM) and lesions) of 27 MS patients identified lower levels of CD4^+^ T lymphocytes in comparison to CD8^+^ T lymphocytes in all sample groups. The highest proportions of CD4^+^ T cells were found in peripheral blood (>30% of total CD3^+^ T cells), then CSF (>20%), and similar numbers in NAWM and lesion (approximately 10% in both). On the other hand, proportions of CD8^+^ T cells were ~50–60% of total CD3^+^ numbers, and did not vary greatly between the regions examined (90).

Flow cytometric analysis of peripheral blood from MS patients and healthy controls demonstrated that MS patients have significantly lower levels of CD4^+^ T lymphocytes and CD3^+^ T lymphocytes, but significantly higher levels of NK T cells (CD3^+^CD16^+^CD56^+^) (91). Moreover, relapsing-remitting MS patients not taking a disease-modifying therapy, with an expanded disability status score (EDSS) of 3 or higher, had significantly higher numbers of CD4^+^IFN-γ^+^ T lymphocytes and CD8^+^IFN-γ^+^ T lymphocytes in peripheral blood in comparison to healthy controls and MS patients with an EDSS below 3 (92). Haegele et al. reported a significant increase in CD8^+^ effector memory T cells in the peripheral blood of patients with MS in comparison to healthy controls, suggesting enhanced immune activation in general. When comparing peripheral blood and the CSF of MS patients, this group reported a significant decrease of CD4^+^ central memory T cells and CD8^+^ effector memory T cells in the CSF compared to blood (93).

Although the level of Treg circulating in the blood are comparable in MS patients and healthy controls, it has been reported that Treg suppressive capacity is reduced in MS and that Treg have lower levels of FoxP3 expression (94–97). Furthermore, when comparing the CSF of patients with other neurological disorders to MS patients, the MS patient group has significantly lower numbers of Treg (97). These findings suggest functional impairment of Treg in MS and that trafficking to the CNS may also be altered in this disease setting.

The role of T cells in CNS regenerative responses has been investigated in settings of experimental CNS demyelination which bear relevance to MS. Using the mouse model of lysolecithin (LPC)-induced demyelination of the spinal cord, Bieber et al. (98) demonstrated that remyelination was impaired in *Rag1*^−/−^ mice that lack T and B lymphocytes. They further showed that when only CD4^+^ T cells or only CD8^+^ T cells were depleted, remyelination was also reduced with greater impact of CD4^+^ T cell depletion. Although this study did not show specificity in the subsets of immune cells influencing this regenerative process, it did bring to light that overall immune suppression may have negative effects on tissue regeneration in settings of demyelination such as MS.

El Behi et al. (99) investigated the differences between regenerative influences of lymphocytes from healthy donors with lymphocytes from MS patients grafted into the spinal cord of nude mice 2 days post-LPC-induced lesioning. Remyelination was significantly lower in mice that had cells grafted from MS patients in comparison to those with cells from healthy controls. This group went on to show that MS lymphocyte supernatants increased the M1/M2 ratio in pure microglial cultures *in vitro*. Conditioned media were collected from these microglial cultures, and media from microglia exposed to MS patient lymphocytes caused an increase in oligodendrocyte progenitor cell (OPC) proliferation but a decrease in OPC differentiation in comparison to media from microglia exposed to healthy control lymphocytes. This would suggest that in MS, CNS-infiltrating T cells could potentially skew the resident microglia phenotype to negatively influence oligodendrocyte-mediated myelin regeneration.

Other studies have sought to elucidate the role of specific subsets of CD4^+^ T cells in remyelination. To determine the effect of Th17 cells in remyelination, mice were fed with the copper chelator, cuprizone, for 4 weeks to induce demyelination and Th17 cells were adoptively transferred when the diet was switched back to normal chow. Two weeks later, mice that received Th17 cells showed significantly impaired remyelination in the corpus callosum as demonstrated by Black-Gold II staining and electron microscopy (100).

On the other hand, Dombrowski et al. (101) investigated the role of Treg in mouse models of myelination and remyelination. When Treg were depleted in Foxp3-DTR mice with LPC-induced spinal cord or cuprizone-induced demyelination, remyelination was significantly impaired. Interestingly, adoptive transfer of Treg rescued this deficiency in the LPC-induced demyelination model. Treg-conditioned media were also shown to significantly increase myelination and remyelination in *ex vivo* brain stem slice cultures, and significantly increase oligodendrocyte differentiation in both murine mixed glia and pure OPC culture models (101). These findings demonstrated a novel direct regenerative role of Treg in the CNS.

The impact of human T cells on human oligodendroglia has also been investigated. *In vitro* experiments using human fetal OPCs showed that Th1 cell supernatants had a cytotoxic effect on OPCs and caused a reduction in the number of O4^+^ cells. Astrocyte-conditioned media from astrocytes exposed to Th1 cells also resulted in the same cytotoxicity and reduction in O4^+^ cells effect. Th2 cells were also studied in these experiments, but showed no significant effect on cytotoxicity or numbers of O4^+^ cells in either systems (102). This suggests that Th1 cells have a negative effect on oligodendrocyte differentiation and can cause cell death through an interaction with astrocytes, however the findings have not yet been tested in *in vivo* models of remyelination.

**Figure 4:**
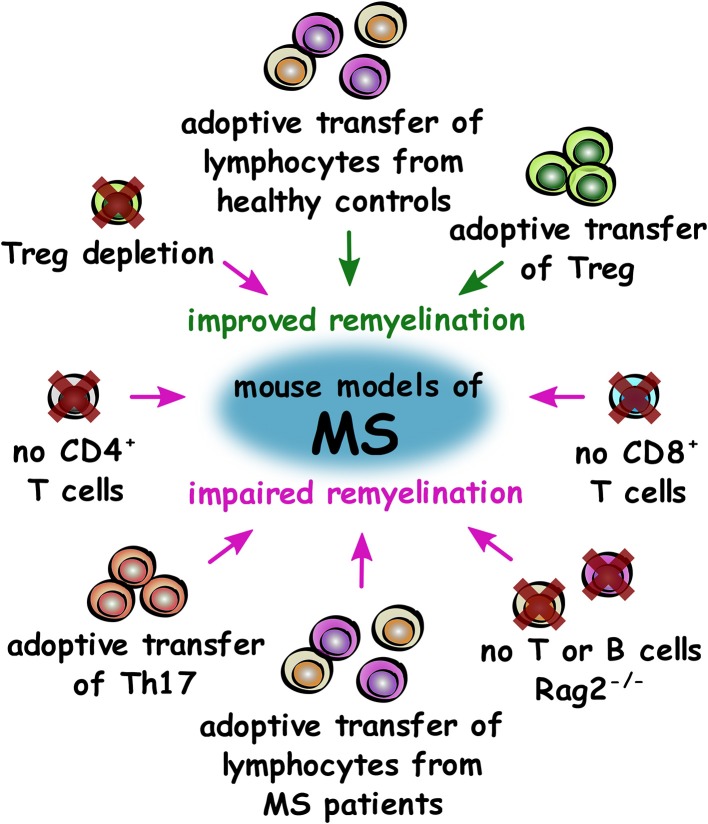
Roles of T cell subsets in improving or impairing remyelination in mouse models of MS. Summary of changes to the adaptive immune system that either improve (green) or impair (magenta) remyelination in mouse models of MS.

Overall, research in experimental models of MS so far demonstrates that the adaptive immune system is required for efficient remyelination in general. Studies have demonstrated that Treg have a pro-regenerative, beneficial role in remyelination, whereas Th17 and Th1 appear to inhibit remyelination and oligodendrocyte differentiation, respectively (Figure 4). Studies on more subsets of immune cells from the adaptive immune system will help to further elucidate these effects and determine if immune mechanisms may offer potential for regenerative treatments for patients.

## CNS Trauma and Injury

Traumatic brain injury (TBI) is a neurological consequence of external forces to the brain, spinal cord or body. Trauma can be separated into a primary and secondary injury: the primary injury is mediated by physical force and causes mechanical tissue deformation that leads to necrotic cell death and damage to blood vessels, neurons and glia. This then triggers subsequent pathological molecular mechanisms described as the secondary injury involving amino acid release and calcium influx, and subsequent increase in free radicals, cytokines, and chemokines, mitochondrial damage, and gene expression changes. Outcomes of this injury frequently include functional neurological deficits due to vascular damage and neuronal and glial cell death (103).

Such aggressive tissue damage will induce a robust inflammatory response and appropriate regulation of this response is critical to stabilization and tissue regeneration. As such, it is likely that Treg would play a key role in this setting. A clinical study by Li et al. (104) found no significant difference in the levels of circulating Treg between controls and TBI patients, however the levels of circulating Treg were found to be significantly higher in survival vs. non-survival TBI patients. Therefore, there appears to be a correlation with the levels of circulating Treg and neurological recovery after TBI and this has been explored further using animal models.

One approach to study the role of the adaptive immune system in TBI used the controlled cortical impact (CCI) model of TBI in mice in which air or an electromagnetic-driven piston is used to penetrate the brain at a known distance and velocity (103, 105). In this model, mice treated with fingolimod, which inhibited T cell trafficking to the CNS, had decreased T and NK cell infiltration, but a higher proportion of Treg present in the CNS, as well as improved neurological functions on the rota-rod and MWM, alleviated brain edema and BBB damage (106). Although this effect may be indirect, it appears that modulation of the immune system has a positive effect on TBI outcomes.

In female mice with optic nerve injury, removal of the deep cervical lymph nodes after nerve injury reduced retinal ganglion cell survival and Treg depletion exacerbated neurodegeneration. To test whether increasing Treg numbers would have a beneficial effect, all-trans retinoic acid was administered to stabilize Treg, enhance Treg suppressive capacity, and increase Treg differentiation. Surprisingly however, this also resulted in exacerbated neurodegeneration (107). This study showed that worsening of neurodegeneration was observed in the presence and absence of Treg, suggesting that Treg may have divergent functions in different phases or types of TBI.

In a gain-of-function study, administration of MBP-specific T cells enhanced retinal ganglion cell survival after optic nerve crush injury in rats (108). Transfer of MBP-specific T cells protected injured neurons in the rat CNS from secondary degeneration due to an increased accumulation of microglia/macrophages and B cells, as well as a transient increase in neurotrophic factors, demonstrating “protective autoimmunity” in this setting (109). Later studies investigated if this mechanism was also functional without the induction of autoimmunity by using glatiramer acetate. Adoptive transfer of T cells reactive to glatiramer acetate in conjunction with glatiramer acetate treatment is also neuroprotective in optic nerve crush injury in rats (110), and treatment of rats with increased intraocular pressure with glatiramer acetate was protective of retinal ganglion cells only in the presence of T cells (111). In a model of rat spinal cord injury (SCI), transfer of MBP-activated T cells or immunization with Nogo-A and other myelin peptides prior to SCI improved recovery. This was evident as higher locomotor function score, improved tissue preservation observed through MRI, higher survival of motor neurons in the ventral horn, more residual myelination (luxol fast blue staining), and higher numbers of myelinated axons (toluidine blue staining and qualitative electron microscopy) (112–115). Tissue repair correlated with T cell accumulation, and T cell-based vaccination with MOG caused promotion of functional recovery after SCI (116). In mice overexpressing the T cell receptor for MBP, retinal ganglion cell survival was increased after optic nerve crush, whereas rats subjected to a thymectomy at birth and therefore lacking T lymphocytes, recovered poorly from this injury. Rats subjected to SCI previously, had higher retinal ganglion cell survival after optic nerve crush in comparison to those not previously subjected to SCI (117) suggesting that prior CNS injury conferred protection to subsequent trauma. After dorsal transection of the spinal cord in mice, vaccination to induce production of polyclonal antibodies to block myelin-associated inhibitors led to regeneration of a large number of axons and partial recovery of motor function. From *in vitro* testing, this was shown to be via promotion of neurite growth (118). A follow-up study comparing immunization to NogoA and myelin-associated glycoprotein (MAG), both of which are found in CNS myelin, also promoted axon regeneration and sprouting (119). These studies suggest that adaptive immune responses to myelin proteins appear to be beneficial in physical trauma in a pro-regenerative manner.

MHC II^−/−^ mice contain only a small CD4^+^ T cell population with limited TCR diversity. After optic nerve crush, these mice had less retinal ganglion cells, and after SCI, demonstrated a poorer outcome on a locomotor function scale. Adoptive transfer of WT CD4^+^ T cells into MHC II^−/−^ mice led to higher numbers of retinal ganglion cells after optic nerve crush and improved functional recovery after SCI. IL-4-producing CD4^+^ T cells conferred neuroprotection in both these injury models and IL-4 acted on neurons to induce axonal growth by potentiating neurotrophin signaling (120).

**Figure 5:**
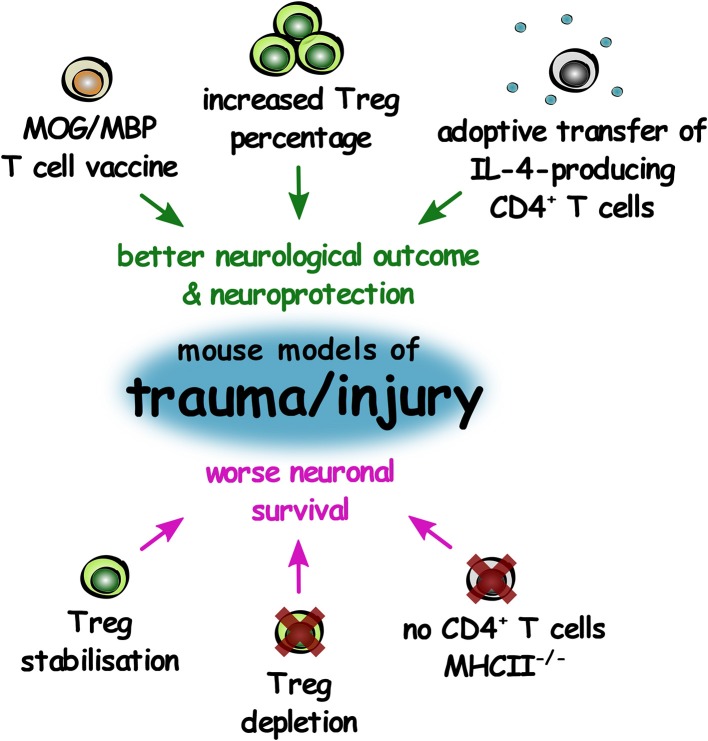
The role of T lymphocytes in improving or worsening outcomes in mouse models of CNS trauma and injury. Summary of changes in T lymphocytes that result in either improved neurological outcome and increased neuroprotection (green), or worse neuronal survival (magenta) in mouse models of CNS trauma and injury.

In physical injury to the CNS, it appears that IL-4-producing CD4^+^ T cells (assumed to be Th2 cells) and interestingly, T cells autoreactive to myelin proteins, exhibit neuroprotective influences. Interestingly, Treg appear to be neuroprotective in TBI, but do not influence the outcomes of nerve crush injury (Figure 5). This may suggest that the role of the adaptive immune response in CNS tissue regeneration can vary based on the type and extent of CNS injury.

## Stroke and Cerebral Ischaemia

Stroke occurs when obstruction or lack of blood flow in the brain causes cell death. Stroke can be ischaemic, where arteries are blocked either by a clot (thrombotic) or plaque accumulation (embolic); or haemorrhagic as a result of excessive bleeding from cerebral blood vessels. One of the most commonly used rodent experimental models to study stroke and cerebral ischaemia is middle cerebral artery occlusion (MCAO). This method involves insertion of an intraluminal filament into the internal carotid artery, blocking middle cerebral artery (MCA) blood flow. This can be transient whereby the filament is removed after a period of time, permanent where the filament is left, or distal MCAO where a craniotomy is performed to cause ligation of the MCA (121).

A number of approaches have been adopted to investigate the role of adaptive immunity in these models. Administration of poly-YE (a high molecular weight copolymer shown to have immunomodulatory effects) facilitated rapid T cell recruitment and activity up to 24 h after MCAO in rats, resulting in improved neurogenesis, reduced neuronal loss, attenuation of behavioral deficits, and improved neurological performance. Poly-YE also resulted in a modulated microglial response through increased IGF-1 production (122). On the contrary, using SCID mice with permanent MCAO, lymphocyte deficiency was associated with reduced neural stem cell (NSC) apoptosis and enhanced NSC proliferation after stroke compared to immunocompetent mice. Depletion of CD4^+^ T lymphocytes in WT mice led to enhanced NSC generation and accelerated functional recovery. However, depletion of CD25^+^ T lymphocytes led to impaired functional recovery and partially impaired neurogenesis, suggesting a key role for Treg in recovery post-stroke (123). It is important to note however, that while CD25 expression is largely associated with Treg in resting conditions, during activation, effector T cells also express CD25.

Expansion of Treg *in vivo* using an IL-2/IL-2 antibody complex lead to significant reductions in infarct size, neuroinflammation, and also led to improvements in motor functions as tested by the rotarod and foot fault test. These beneficial effects were eliminated through depletion of Treg using diptheria toxin in Foxp3-DTR mice. Adoptive transfer of Treg from IL-2/IL-2 antibody complex treated mice also lead to greater neuroprotection in comparison to Treg transferred from mice treated with only isotype control antibody, suggesting an improvement in Treg immunomodulatory function (124). An influx of Treg in the brain are detectable after ischaemic stroke in mice, which significantly improves recovery from neurological deficits. These brain Treg also secrete amphiregulin to subdue astrogliosis and reduce neurotoxicity, providing a mechanism for Treg-mediated neuroprotective effects (125).

Through a range of studies it has become apparent that changes in the infarct size and neurological outcomes vary depending on the component of the adaptive immune system that is modified. Improved neurological functional outcome and smaller infarct size were observed in SCID mice (lacking B and T lymphocytes), MHC II^−/−^ (CD4^+^ T cell deficiency), and JNK2^−/−^ (Th1 differentiation impairment), in comparison to controls in the MCAO model. A smaller infarct size but no neurological functional difference was observed in the *Tap1*^−/−^ mouse model (reduced CD8^+^ cells). A larger infarct size and worse neurological function was observed in IL-4^−/−^ mice (Th2 impairment as well as other subset perturbation), and no significant effect was seen in *Ebi3*^−/−^ (impairment of Treg and other subsets through IL-27/IL-35 deficiency) (126). In particular, depletion of Treg via anti-CD25 treatment increased delayed brain damage and deteriorated functional outcome (forelimb use asymmetry test and corner test). Absence of Treg caused increased activation of pro-inflammatory microglia and T cells, while treatment with IL-10 abrogated the overexpression of inflammatory cytokines (127). Interestingly this is supported by a clinical study, demonstrating that lower IL-10 plasma concentrations in acute ischaemic stroke patients is associated with clinical worsening (128). Another study on human ischaemic stroke demonstrated that there is an increase in BDNF^+^ Treg cells in stroke patients compared to healthy controls, and that those stroke patients with higher percentages of BDNF^+^ Treg in their serum had a better neurological outcome 6 months post-stroke (129). It is important to note the limitations of the genetically altered models listed however as other immune cell subsets will also have been impacted in these models.

Similar to models of CNS trauma, it appears that autoimmunity against myelin proteins is beneficial in mouse models of stroke. Nasal vaccination of mice undergoing MCAO with MOG led to induction of CD4^+^ IL-10-producing T cells, reduction of infarct size, and improved behavioral score (130). A follow-up study demonstrated that adoptive transfer of IL-10-producing MOG-specific CD4^+^ T cells conferred neuroprotection after ischemic stroke (131). MCAO rats tolerized to MBP had improved neurological function and better behavioral outcomes when they had more Treg in response to MBP as opposed to a Th1 pro-inflammatory response to MBP (132, 133).

In mice with intracerebral hemorrhage (in which blood from the tail vein was injected into the caudate nucleus), Treg depletion (using anti-CD25 or Foxp3-DTR mice) led to increased neurological deficit scores and neuronal degeneration. Conversely, when Treg were expanded using an anti-CD28 super-agonist antibody, inflammatory injury was reduced and microglia and macrophages shifted to an M2-like phenotype (134). Previous work by this group had shown that mice with transient MCAO with an activated Treg phenotype had enhanced neurogenesis (proliferation of NSCs) in the subventricular zone (SVZ) after ischaemia (135). Pre-treatment of rats with resveratrol prior to MCAO injury was found to be neuroprotective and associated with lessened neurological deficits and infarct size. This effect was attributed to expansion of Treg in the spleens and ischaemic hemisphere, an increase in IL-10 and reduction in IL-6 and TNF-α in the plasma and ischemic hemisphere (136).

Irradiation with 710 nm visible light has been used as a method to expand the total number of lymphocytes. Rats with MCAO irradiated in this way had improved neurological and step fault scores, decreased infarct sizes, higher Treg immunoreactivity, increased IL-10 mRNA expression and reduced microglial activation (137). Adoptive transfer of Treg into ischaemic rats (from transient MCAO), or expansion of Treg by anti-CD28 super-agonist treatment reduced infarct size at 3–28 days post-insult. However, there was no alteration in the rate of proliferation of NeuN^+^, NCAM^+^, or CD31^+^ cells (138). Similar studies using adoptive transfer of Treg also demonstrated a reduction in inflammatory cytokines and improved immune homeostasis. BBB damage was ameliorated through PD-L1 leading to neurological improvement measured on the five point neurological scale (139–141).

**Figure 6:**
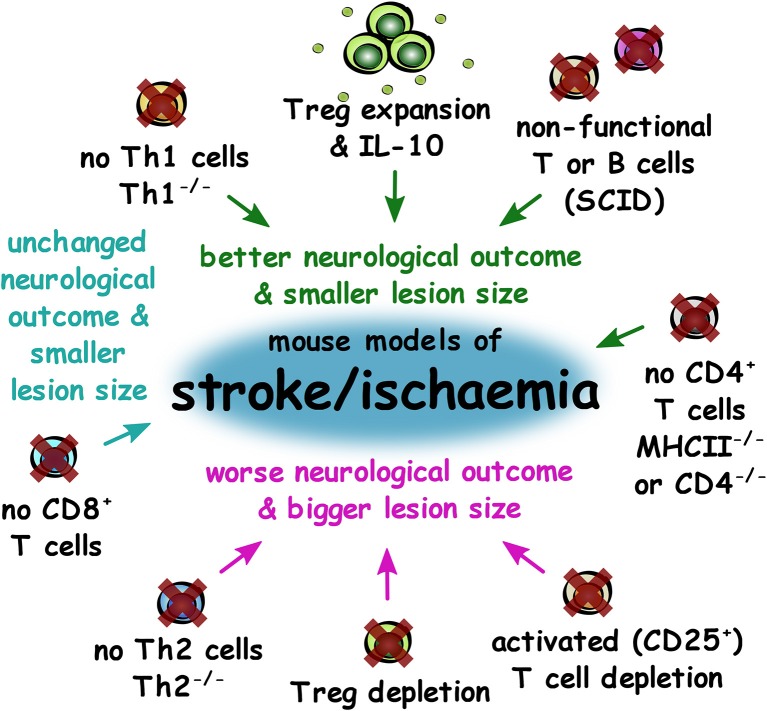
Impact of experimental adaptive immune system changes in outcomes of mouse models of stroke and ischaemia. Summary of changes to the adaptive immune system that result in better neurological outcome and smaller lesion size (green), unchanged neurological outcome, and smaller lesion size (cyan) or worse outcome and larger lesion size (magenta) in mouse models of stroke and ischaemia.

Overall, depletion of components of the adaptive immune system or of total CD4^+^ T lymphocytes led to better neurological outcomes and smaller lesion size in mouse models of stroke, but CD8^+^ T cell depletion alone had no effect. However, Treg depletion led to worse neurological outcomes and larger lesion sizes, suggesting that it is pro-inflammatory CD4^+^ T cells that contribute to poor outcomes in stroke (Figure 6).

## Concluding Remarks

**Figure 7:**
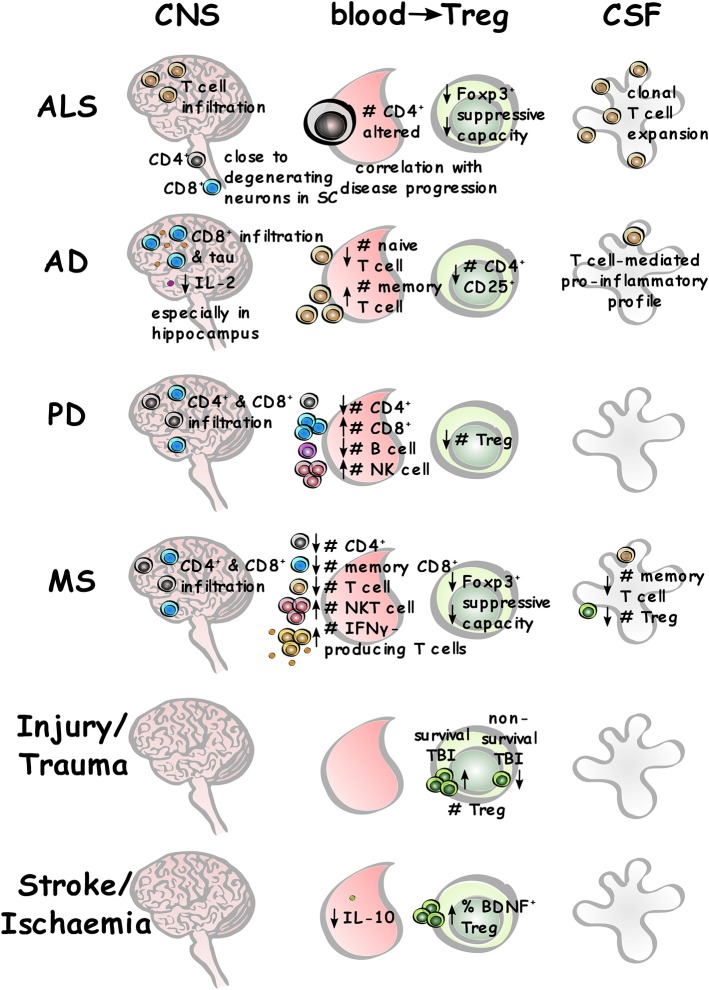
Adaptive immune system changes across different neurological conditions. Summary of different lymphocyte populations in the CNS, blood (with specific Treg differences), and CSF across different neurological conditions where relevant and measurable.

The studies reviewed here contribute key advances to our understanding of the role of CD4^+^ T lymphocytes in pro-regenerative outcomes across a range of neurological conditions. Although low levels of adaptive immune cell infiltration occur in CNS homeostasis, this is increased across neurological disorders. Alterations in peripheral immune cell subsets and functions are observed in the blood of patients with neurological disease, and this will affect the infiltrates present in the CSF and CNS, particularly in diseases with BBB disruption (summarized in Figure 7).

Overall there are no definitive CD4^+^ T cell subsets that are wholly beneficial or wholly detrimental across all the neurological conditions described, with the most conflicting and controversial data arising from mouse models of AD. However, lower levels or function of Treg in peripheral blood appear across all the neurological diseases reviewed, with lower suppressive capacity reported in AD and MS patient samples, and better outcomes in CNS injury when higher levels of Treg are present. Further research into these effects may help to develop better and more specific therapeutic targets, and avoid global modulation of the immune system, which can render patients immunocompromised and susceptible to a range of pathogenic threats. The emergence of regenerative functions of T cells will provide a range of new mechanisms that can be exploited for therapeutic targeting and potentially deliver the benefits of T cells in the CNS without having to deliver T cells to the CNS.

## Author Contributions

FE wrote the manuscript. MD designed and created the figures. FE, MD, AF, and DF contributed to literature research and editing of the manuscript.

### Conflict of Interest Statement

The authors declare that the research was conducted in the absence of any commercial or financial relationships that could be construed as a potential conflict of interest.
